# Medical Journalism as a Career

**Published:** 1913-09-06

**Authors:** 


### MEDICAL JOURNALISM AS A CAREER.

### The Available Openings on the Hospital Press.

Very few medical students and only a small proportion of medical practitioners are aware that such a thing as medical journalism exists. They are all quite familiar with the leading medical journals, but how these are put together and produced, and by whom, are matters upon which they are totally ignorant. It is enough for them that the papers appear week by week.

The idea of this short article is not so much to lift the veil which hides the mechanism of the medical press as to indicate that medical journalism is a specialised vocation of the highest value and interest which calls for special qualities, and to some extent a special training, in those who devote themselves to it. The fact that the world of the medical press is small and circumscribed should not prevent the world of medicine at large from understanding more about the aims and attributes of those who shape professional opinion and chronicle medical events and discoveries. The medical press has nothing to gain from obscurity.

Medical journalists may be divided into two distinct groups, amateurs and professionals, and of these the latter (with whom we are principally concerned) may be subdivided into those who devote themselves entirely to writing and editing, and those who combine journalism with the practice of their profession. The amateurs range from students who conduct with varying ability the hospital gazettes to leaders of the profession, whose enthusiasm for some special periodical takes toll of their means and leisure. Speaking generally, most of those who enter seriously upon whole-time medical journalism do so after some years' experience in a part-time capacity. It used to be said that all journalists "drifted" into Fleet Street. This is probably now true of only a small minority of lay pressmen, but the higher ranks of medical journalism are still recruited for the most part from the active list of medical practitioners. Very few, if any, medical students enter medicine with the intention of passing into medical journalism. Those who eventually do so are always men with a stronger bent for literature than for practice, but the decision is seldom made until some years after qualification, and then only as the result of long and anxious thought. For medical practice is a jealous mistress, and the man who renounces her for journalism must first make very sure of his fitness for the new life and of his prospects in it.

The subordinate posts on the medical press are not, of course, filled by medical practitioners nor by those who have had any direct medical training; nevertheless, the men who hold these positions are as a class remarkable for their efficiency and for their accurate appreciation of technical phrases and terms. The higher editorial posts are of necessity almost exclusively open to men trained in medicine and intimately acquainted with the various phases of professional life in hospital and private practice. Without this training and experience they would lack the essentially professional outlook and the critical judgment of medical and scientific questions which are indispensable in the medical editor.

While he cannot have too wide a knowledge of medicine and of medical practice, the medical editor need not have expert knowledge of any one branch of professional work. Indeed, it may be better for the preservation of his editorial balance if he has never identified himself with a specialty and is willing to admit ignorance of detail to each member of his expert advisory staff. But while a certain air of detachment is valuable, the editor must keep an open mind for new ideas and methods and a sympathetic, though guarded, ear for enthusiasts.

Certain innate qualities are desirable in the medical journalist. In a short article intended primarily to interest students of medicine in a subject which will not otherwise be brought to their notice, it would be unwise to attempt detailed treatment. The medical press offers a full and attractive life to the man whose tastes and abilities incline towards the literary and political aspects of medicine. No student or practitioner who lacks the journalistic instinct should press for further particulars about a sidepath of medicine in which he will certainly come to grief. The world of medical journalism is small and its material rewards are slender; only those with a strong bent and proved aptitude should turn to consider its claims.

Having thus discouraged those who are unsuited to the life we will conclude with a few further observations on the mental equipment which should be possessed by the medical journalist. Assuming a sound general training in medicine and surgery, he should have the faculty for rapid assimilation of fresh topics and a gift of ready expression. While a strong taste for writing and a sense of literary style are extremely important assets in those who write for a medical journal and assist in its editorial conduct, the highest posts call for certain qualities in addition. In the medical editor a keen insight into men and affairs and a level head are at least as essential as a critical ear for the English language. He must be able to go rapidly to the heart of a subject, to sketch out the heads into which it naturally falls, and to indicate these in a few pregnant words to the most appropriate writer upon his staff. He must combine in himself breadth of view, initiative, and a vigilant eye for detail. This combination is rare, but every one of the qualities which go to the making of a successful medical editor can be strengthened and perfected during the inevitable years of apprenticeship. The days of combative medical journalism, when the first Thomas Wakley laid the foundations of the *Lancet*, are long past, and although enterprise and courage are still vitally necessary, other attributes, such as tact, foresight, and prudence, are now equally important. The medical press of this country has gradually built up a world-wide reputation for integrity and solid worth, and something more than ordinary ability is called for from those who aspire to carry on its traditions.

